# Physiopathological mechanisms underlying Alzheimer’s disease: a narrative review

**DOI:** 10.1590/1980-5764-DN-2024-VR01

**Published:** 2024-12-16

**Authors:** Eliasz Engelhardt, Elisa de Paula França Resende, Karina Braga Gomes

**Affiliations:** 1 Universidade Federal do Rio de Janeiro, Instituto de Neurologia Deolindo Couto, Rio de Janeiro RJ, Brazil.; 2 Universidade Federal de Minas Gerais, Faculdade de Medicina, Belo Horizonte MG, Brazil.; 3 Faculdade Ciências Médicas de Minas Gerais, Belo Horizonte MG, Brazil.; 4 Universidade Federal de Minas Gerais, Faculdade de Farmácia, Belo Horizonte MG, Brazil.

**Keywords:** Alzheimer Disease, Amyloid Plaque, Amyloid, Neurofibrillary Tangles, Tau Proteins, Biomarkers, Doença de Alzheimer, Placa Amiloide, Amiloide, Emaranhados Neurofibrilares, Proteínas Tau, Biomarcadores

## Abstract

The neuropathological signature of Alzheimer’s disease (AD) comprises mainly amyloid plaques, and neurofibrillary tangles, resulting in synaptic and neuronal loss. These pathological structures stem from amyloid dysfunctional metabolism according to the amyloid cascade hypothesis, leading to the formation of plaques, and apparently inducing the initiation of the abnormal tau pathway, with phosphorylation and aggregation of these proteins, ultimately causing the formation of tangles. In this narrative review, the existing hypothesis related to the pathophysiology of AD were compiled, and biological pathways were highlighted in order to identify the molecules that could represent biological markers of the disease, necessary to establish early diagnosis, as well as the selection of patients for therapeutical interventional strategies.

## RESUMO

A assinatura neuropatológica da doença de Alzheimer (DA) compreende principalmente placas amiloides e emaranhados neurofibrilares, resultando em perda sináptica e neuronal. Essas estruturas patológicas decorrem de um metabolismo amiloide disfuncional, conforme a hipótese da cascata amiloide, levando à formação de placas e aparentemente induzindo o início da via anormal do metabolismo da proteína tau, com fosforilação e agregação dessas proteínas, culminando na formação dos emaranhados. Nesta revisão narrativa, as hipóteses existentes relacionadas à fisiopatologia da DA foram compiladas, e as vias biológicas foram destacadas para identificar as moléculas que poderiam representar marcadores biológicos da doença, necessários para estabelecer o diagnóstico precoce, bem como a seleção de pacientes para estratégias de intervenção terapêutica.

## INTRODUCTION

The neuropathological signature of Alzheimer’s disease (AD) comprises mainly “amyloid plaques” (senile plaques) and “neurofibrillary tangles” (NFTs), accompanied by an inflammatory process, microglial and astrocytic activation, and finally by synaptic and neuronal loss^1,2,3,4^.

This neuropathological knowledge was obtained at the beginning of the 20^th^ century by Alois Alzheimer^5^ and Oscar Fischer^6^, and the definite diagnosis of AD began to be based on brain biopsy *in vivo* (purposely or incidentally)^7,8,9^ or on *postmortem* examination of the brain^9,10^.

The resulting research that followed these pioneer studies, conducted over subsequent decades, clarified the pathophysiological steps, and the components that generate the neuropathology (e.g., amyloid and tau pathology)^11,12^. This knowledge, comprising the formation, localization, and clearance of the components of the pathological structures, permitted the development of laboratory techniques to detect them in body fluids, such as cerebrospinal fluid (CSF) and blood^13^, and through molecular imaging techniques^14^, in order to obtain their normal and abnormal patterns. Such laboratory and imaging findings constitute what are referred to as “biological markers” of the disease. These markers represent tools that permit establishing the diagnosis *in vivo* in the early stages of the disease, or even in the preclinical stage, allowing for the proper selection of subjects for clinical trials and for emerging treatment strategies^15^.

This narrative review is focused on the multiple characteristics of the processes that give rise to these biological markers.

## THE AMYLOID PATHOLOGY

The main component of the plaques are amyloid peptides, and the pathophysiology of AD, presently, considers chiefly the amyloid formation as the primary cause of the disease, constituting the “amyloid cascade hypothesis”^16,17,18,19^.

The amyloid cascade hypothesis postulates that a change in the large “amyloid precursor protein” (APP) molecule processing, increasing amyloid production or decreasing its clearance rates, causes extra-neuronal aggregation of amyloid peptides, and the formation of amyloid plaques. This process is accompanied by microglial and astrocytic activation, local inflammatory responses, and oxidative stress, and followed by tau protein changes, which result in the formation of intraneuronal NFTs^20,21,22^.

### Amyloid Precursor Protein characteristics and processing

**Figure 1:**
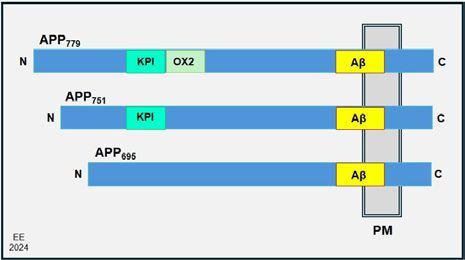
Amyloid Precursor Proteins isoforms, with three different lengths.

Abbreviations: APP, Amyloid Precursor Protein, N, N terminus; C, C terminus; KPI, Kunitz protease inhibitor; OX-2, 0X-2 domain (insert of 19 amino-acid residues that is similar to a region of the Ox-2 antigen, commonly found in cell-surface receptors, involved in cell-surface binding and recognition); Aβ, amyloid-β peptide segment; PM, plasmatic membrane.

The APP molecule is a type I transmembrane glycoprotein, consisting of a single-pass transmembrane domain, with a large extracellular (luminal) amino terminus (N terminal) (ectodomain), and a short carboxyl cytoplasmic (cytosol) terminus (C terminal). This protein constitutes a family of different isoforms that are generated by the *APP* gene localized in chromosome 21 (band 21q21)^23,24,25,26^. The molecule appears under three isoforms (APP770, APP751, APP695). The isoform APP770 is the longest and contains a Kunitz-type proteinase inhibitor (KPI) domain and an insert of 19 amino acid residues (OX-2) domain (involved in surface cell-binding and recognition); the APP751 contains only the KPI domain; and the APP695 does not contain the mentioned domains. These three isoforms possess the amino acid sequence of the amyloid peptide^16,24,26,27,28^ (Figure 1).

The APP695 molecule is expressed prominently in the brain and mostly found in neurons, whereas the APP751 and APP770 isoforms are expressed at considerably lower levels, found mostly in astrocytes and peripheral cells, including the platelets^16,23,26,27,28^.

The proteolytic processing (cleavage) of the APP molecule, in normal and pathological conditions, follows two concurrent pathways, one major, non-amyloidogenic, and another minor, amyloidogenic, performed by specific proteases (the secretases α-, β-, and γ-)^26,29,30^. The α-secretase cleavage is performed by two molecules of the ADAM family (disintegrin and metalloproteinases — ADAM-10 and ADAM-17 or TACE), which slice the APP molecule in the ectodomain within a sequence known as amyloid-β (Aβ), precluding the formation of the Aβ peptide, and thus related to the non-amyloidogenic pathway. The β-secretase cleavage is performed by two molecules BACE2 and mainly BACE1, which may slice at two sites of the ectodomain, one of them at the border of the ectodomain Aβ sequence, while the other cleaves distally. Finally, the γ-secretase complex, formed by the presenilin (catalytic subunits of PS1 or PS2), and three partner proteins (Aph-1, Pen-2, and nicastrin) cleave the intramembrane segment of the APP molecule in multiple sites, contributing, if occurring after the β-secretase cleavage, to the formation of Aβ peptides of varied lengths (mainly Aβ40 or Aβ42). The γ-secretase cleavage occurs after the completion of either α- or β-secretase activity^30,31,32,33^.

**Figure 2:**
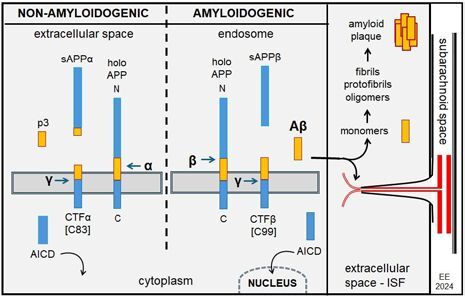
Amyloid precursor protein cleavage of non-amyloidogenic and amyloidogenic pathways.

Abbreviations: holoAPP, entire Amyloid Precursor Protein; N, amino (NH2) terminus; C, carboxy (COOH) terminus; sAPPα, soluble APPα; sAPPβ, soluble APPβ; CTFα, carboxy-terminal fragment α (83-amino acids C-terminal fragment); CTFβ, carboxy-terminal fragment β (99-amino acids C-terminal fragment); p3, 24, or 26 residue peptides; AICD, amyloid precursor protein intracellular domain; Aβ, amyloid-β peptide; ISF, interstitial fluid.

Notes: Extracellular space: locale where released Aβ monomers undergo fibrilization and formation of plaques; ISF: place through which Aβ peptides reach the clearance pathways.

The APP molecule cleavage follows certain sequences, being first cleaved within the ectodomain by α-secretase or β-secretase, resulting in the shedding of almost the entire ectodomain as a soluble fragment sAPPα (with part of the amyloid segment), and a membrane-bound α-C-terminal fragment (α-CTF or C83) (with part of the amyloid segment), or a soluble fragment sAPPβ (without part of the amyloid segment), and a β-fragment (β-CTF or C99) (with the entire amyloid segment), respectively. The latter fragments are subsequently cleaved at several adjacent sites of the intramembrane (transmembrane) domain by γ-secretase to release a p3 peptide (non-amyloidogenic) from C83, or the Aβ peptides (amyloidogenic) from C99, into the extracellular milieu. The amyloidogenic process produces varied monomers, including the Aβ40 and Aβ42 ones, which are neurotoxic and capable of oligomerization, aggregation, and subsequent plaque formation. Almost 90% of secreted Aβ ends in residue 40 (Aβ40), whereas that ending at residue 42 (Aβ42) accounts for less than 10% of secreted Aβ. The Aβ42 monomer is the major, and sometimes the only component in amyloid plaques, capable of oligomerization, aggregation, and subsequent plaque formation, while Aβ40 is several-fold more abundant in the brain. The γ-secretase cleavages generate, in both pathways, a cytoplasmic polypeptide termed AICD (APP intracellular domain). Additionally, minor amounts of other forms (lengths) of Aβ peptides have also been detected^23,26,29,30,31,34^ (Figure 2).

### Amyloid precursor protein trafficking and release

The APP molecule is synthesized in the endoplasmic reticulum, and transported to the Golgi complex, where its maturation is completed, finally reaching the plasma membrane or the endosomes. The mature APP is cleaved at the plasma membrane mainly by α-secretase, followed by a γ-secretase cut, forming fragments without the amyloidogenic peptide, whereas the endosomal APP is cleaved by the action of the β- and γ-secretase, producing Aβ peptide and other fragments. The Aβ generated is, in part, released into the extracellular space and, in part, associated with the plasma membrane. The endocytosed Aβ has also access to other subcellular compartments through the vesicular transport system^23,35^.

### Amyloid Precursor Protein and Alzheimer’s disease

The understanding of the central role of Aβ in AD pathogenesis was obtained through studies of the inherited (familial) types of the disease, including pathogenic *APP*, *PS1*, and *PS2* gene mutations, which account for about 1% of all AD patients, but which may be seen as model forms^30,36,37^. Additionally, trisomy of chromosome 21 (as in Down syndrome), where the *APP* gene is located, and the rare hereditary condition of “locus duplication” are also causes of AD^38,39^.

The sporadic form of AD responds for the majority of disease cases and is associated to several risk genes. The “genome-wide association studies” (GWAS) have revealed over 130 AD-associated susceptibility genes, whereas “whole genome sequencing” (WGS) and “whole exome sequencing” (WES) studies have identified many new AD-associated rare variants. These risk genes include apolipoprotein-E-ε4 (*APOEε4*) allele, and a large number of variants in other genes, some of them also present in familial cases. The *APOEε4* increases Aβ deposition by promoting its production and fibrillization and impairing degradation-clearance pathways. Additionally, it increases the accumulation of tau pathology by intensifying its phosphorylation and aggregation. Most risk genes control pathways are related to the production, trafficking, and clearance of Aβ. Besides the genetic influence, the sporadic AD form is also influenced by environmental, modifiable lifestyle, and vascular risk factors^30,40^.

### The amyloid-β peptides – further changes

The Aβ peptide is a normal product derived from the APP cleavage process. It is an amino acid sequence of diverse lengths, further cleaved to the main final forms, mainly the 40-amino acid Aβ40 and the 42-amino acid Aβ42 monomers. The Aβ monomers can form various types of assemblies, from small aggregates of 2 to 12 peptides “oligomers” to “protofibrils” and “fibrils”. The oligomers that may normally be embedded in the membrane bind to transition metals such as Cu, Zn and Fe. The amyloid fibrils are large and insoluble, and they can further assemble into amyloid plaques, forming the histological lesions that are characteristic of AD, while amyloid oligomers are soluble and may spread throughout the brain. The fibrils of Aβ form a parallel, in-register cross β-sheet structure^23^. The Aβ peptides oligomers produce diffusible ligands (Aβ-derived diffusible ligands [ADDLs]) before forming fibrils^41^.

Considering physiological conditions, most Aβ peptides are found in the form of Aβ40 while less than 5% of newly generated Aβ form the Aβ42 peptide. The Aβ42 is more susceptible to aggregation than Aβ40 and to initiate formation of pathological oligomers, fibrils, and plaques^42^.

### The amyloid-β peptide degradation and clearance

The production of Aβ is normally balanced by several processes, involving degradation, clearance, transport out of the brain, and deposition into insoluble aggregates. Some studies suggest that proteolytic degradation is a significant cause of brain Aβ levels, and therefore, of Aβ-associated pathology. The aging-associated reduction of catabolism is a strong candidate mechanism that could account for the accumulation in the aged brain. The Aβ-degrading proteases neprilysin, endothelin-converting enzymes, insulin-degrading enzyme, plasmin, and other proteases play important roles in amyloid degradation and AD^23,43^.

Additionally, Aβ peptides that are released into the extracellular space can be transported between different compartments, from the brain to the blood, and from the blood to the brain; it can also be cleared by chaperones, as ApoE, which can affect Aβ metabolism after it is released by cells, and influence Aβ aggregation, clearance, and transport^23,37,43^.

There is evidence suggesting that the “lipoprotein receptor-related protein” (LRP) and the “receptor for advanced glycation end products” (RAGE) are involved in receptor-mediated flux of Aβ across the “blood-brain barrier” (BBB). There is also indication that 10–15% Aβ can enter the CSF from the brain’s “interstitial fluid” (ISF) and then into the blood circulation. Other studies indicate that Aβ can be transported across the BBB and cleared from the brain after binding to LRP. Thus, the carrier- and receptor-mediated transport of Aβ across the BBB appears to contribute to the regulation of brain Aβ levels^23,37,44^.

Studies demonstrate the importance of vascular transport across the BBB in clearing Aβ from the brain into the circulation. This clearance mechanism is age-dependent, and lower clearance rates in older individuals correlate with decreased vascular abundance of LRP. Data support the concept that the vascular system plays an important role in regulating the levels of Aβ in the brain. The findings further suggest that if the levels of Aβ in brain extracellular space exceed the transport capacity of the clearance mechanism across the BBB or if the vascular transport of the peptide were impaired, this would result in the accumulation of Aβ in the brain, and possibly the formation of amyloid plaques. Vascular transport out of the brain across the BBB may represent a major physiological mechanism that prevents accumulation of Aβ and amyloid deposition in the brain^23,44^.

### The plaques formation, types, and characteristics

The Aβ peptides, after their generation (monomers), are released into the extra-neuronal space, where they can aggregate in oligomers of various sizes and flow in the ISF. Besides, they may react with receptors on neighboring cells and synapses, affecting their function^23^. Some of these oligomers are cleared from the brain. Those that are not cleared out clump together with more segments of Aβ. As more segments are grouped, the oligomers become larger, forming protofibrils and fibrils. The fibrils link with other protein molecules, neuron terminals, and glia, and form the “plaques”. Inspection of the Aβ peptide isoforms in the amyloid fibrils revealed that they predominantly contained Aβ42, and less abundantly Aβ40^23,45,46,47^. The Aβ peptide is released from neurons into the extracellular space modulated by synaptic activity, both pre- and post-synaptically^45,46^.

The plaques are primarily composed of Aβ peptides but vary in their composition. A large number of other compounds have been colocalized with Aβ in the plaques of AD brains, such as proteoglycans, complement proteins, ApoE, α2-MG, IL-1α, IL-6, and α2-MGR/LRPR, with lipids, metal ions, reactive oxygen species and nucleic acids amyloidogenic related molecules, protease and clearance related elements, in addition to antioxidant proteins^41,44,46,47,48,49^.

The Aβ plaques are extracellular formations of approximately spheroid shape, containing Aβ peptides with a β-pleated sheet conformation, associated with other molecules. A gradual growth in the interstitial space of the brain through continual extracellular deposition of Aβ peptides at “seeding sites” is seen, these growing plaques encroach increasingly on neurons and their axons and dendrites processes, finally leading to neuronal death^50^. The plaques are seen as three types: “diffuse plaques”, “dense-core plaques”, and “neuritic plaques”^2,41,46,51^.

The diffuse plaques are formed by extracellular Aβ deposits of varying sizes and are caused by outflows of amyloid from blood vessels at focal sites of BBB openings. They comprise from very small, often stellate assemblies scattered in the parenchyma, to confluent, sometimes large patchy accumulation, at times in the subpial cortex, as large “cotton-wool plaques”. The diffuse amyloid plaques are present in normal aging and in cognitively normal individuals, being numerous in AD. Such plaques lack neuritic components, as well associated inflammatory cells^2,41,46,50,51^. There is a consensus that diffuse plaques are the earliest type to appear, followed later by cored ones^46^.

The dense-core plaques are characterized by the presence of a central condensed core of amyloid with a β-pleated sheet configuration, surrounded by a clear region with scarse Aβ surrounded by an outer corona of more diffuse Aβ. The relatively clear intermediate space and the outer corona are occupied by neuronal and glial elements. The compact reticular or radiating dense amyloid suggests the presence of more fibrillogenic forms of Aβ, which includes the ability to attract inflammatory cells^2,41,46,50,52^.

The neuritic plaques (NPs) represent a subset of dense-core plaques, and consist of a dense center surrounded by a clear halo, with numerous threads of amyloid radiating towards the periphery, surrounded by a corona of abnormally formed neurites (neuronal processes — both dendrites and axons), under the form of dystrophic processes filled with paired helical filaments (see below). This kind of plaques are thought to be more closely associated with neuronal loss in AD. Considering the disruption of the structure and trajectory of neuronal processes, they are believed to interfere with the connectivity and network functionality of the brain. Many different neurotransmitter systems contribute to anomalous neurites, and individual plaques can contain neurites from multiple sources. In addition, NPs are generally more strongly associated with dementia than diffuse plaques. Synaptic pathology is especially evident in the immediate vicinity of Aβ plaques, and the loss of synapses correlates strongly with the degree of dementia in AD. These plaques frequently contain, at the periphery, activated microglia cells and reactive astrocytes related to an inflammatory process^2,50,46,51,52,53,54^.

### Topography of cerebral amyloid

AD is characterized, as seen, by histopathologic lesions comprising plaques and tangles^55^. The former is one of the components of the AT(N) classification, accepted for the description of multidomain biomarker findings for amyloid (A), tau (T), and neurodegeneration (N)^56,57^.

The knowledge of the distribution patterns of the plaques is of great importance for a better understanding and interpretation of Aβ-PET images^14^.

The distribution pattern, in the Braak and Braak staging of amyloid, was obtained through immunostaining with antibodies against A4 amyloid protein (Aβ40). The cerebral cortex, in particular the isocortex (neocortex), is the favorite site for the initial deposition of amyloid. Most plaques remain devoid of pathologically changed neurites, and show neither distortions of the neuropil nor accumulations of glial cells^58^ (Box 1).

**Box 1. Staging of amyloid spreading according to Braak and Braak^58^.**

**Stage A.** Isocortex basal parts (frontal, temporal, and occipital lobes): low-density amyloid deposits; presubiculum and entorhinal cortex: weakly stained amyloid.

**Stage B.** Isocortical association areas (mainly basal parts of frontal, temporal, and occipital lobes): medium-density deposits in almost all areas; frontal and parietal lobes contiguous to the central region: scattered deposits; primary sensory and motor areas: absent or small deposits; hippocampal formation: mildly involved; white matter underlying the cortex: amyloid found.

**Stage C.** Isocortex: dense amyloid deposits in practically all areas; primary sensory and motor areas: amyloid deposits present; hippocampal formation: relatively few amyloid deposits.

**Subcortical structures.** Striatum, thalamus, hypothalamus, subthalamic, and red nuclei: gradual deposition of amyloid; SNpc: almost devoid; cerebellar cortex: patches of amyloid may be present.

Abbreviation: SNpc, substantia nigra pars compacta.

Evidence shows that Aβ propagates from one brain region into others by following neuronal connections. The progression of AD is also accompanied by changes in the composition of Aβ plaques — a sequence described as Aβ maturation stages. A prion-like seeding process is apparently one of the mechanisms underlying the induction and propagation of the Aβ pathology in the brain^59^. Such a feature is seen in the staging provided by Thal et al., considering the distribution of Aβ deposits changes with time, and reflecting the course of the expansion of the amyloid pathology in the brain, as observed by immunostaining of Aβ and the Gallyas silver staining. The Aβ deposition begins with diffuse plaques; at later phases, other types develop such as cored plaques and NPs^4,60^ (Box 2).

**Box 2. Staging (phases) of progressive Aβ deposition according to Thal et al.^41,60^.**

**Phase 1.** Neocortex (frontal, parietal, temporal, occipital): sparse, small groups of diffuse plaques present.

**Phase 2.** Allocortex (entorhinal region, subiculum, hippocampus [CA1], cingulate), and amygdala: plaques presente.

**Phase 3.** Basal ganglia (caudate, putamen), thalamus, and hypothalamus: become involved.

**Phase 4.** Midbrain (including SNpc and red nucleus), and medulla oblongata: amyloid deposits present.

**Phase 5.** Pons, and cerebellum: involved.

Abbreviation: SNpc, substantia nigra pars compacta.

### Amyloid as biomarker for Alzheimer’s disease

The amyloid peptide levels are elevated in the brain, but Aβ42 is reduced in body fluids (blood plasma and CSF) of patients with AD, being measurable at these sites. Although the individual amount of each of the main forms in the CSF, Aβ42, and Aβ40 may be of some meaning, the ratio Aβ42/40 has been suggested to be superior in discriminating patients with AD, because it is a method of normalize the Aβ42 concentration for the total Aβ concentration, represented by the most abundant Aβ40 monomer. In addition, the Aβ42/40 ratio has been shown to improve the accuracy of the differential diagnosis of AD from other dementia disorders. The Aβ42/40 ratio appears also to be changed in patients with mild cognitive impairment (MCI) who developed AD dementia, compared to cognitively stable MCI patients, and MCI patients who developed other forms of dementia. Improved concordance between Aβ-PET and CSF Aβ was also observed when using the Aβ42/40 (and Aβ42/38) ratios instead of Aβ42 alone^61,62^.

## THE NEUROFIBRILLARY PATHOLOGY

The neurofibrillary pathology comprises mainly “neurofibrillary tangles” (NFTs), “neuropil threads” (NTs), and “dystrophic neurites” (DNs) related to NPs^2,58,63^.

The NFTs are abnormal filamentous bundles that accumulate in neuronal perikarya, dendrites, and axons, well demonstrated in light microscopy with silver staining methods. Studies with electron microscopy demonstrated the structural features of tangles, which are composed of abnormal filaments, referred to as “paired helical filaments” (PHFs), showing a double helical stack of transversely oriented subunits twisted into a ribbonlike structure^64^. They are formed mainly of hyperphosphorylated tau (p-tau) aggregates, which accumulate inside neurons. The PHFs that constitute the tangles are pathological assemblies of proteins, formed by tau proteins (about 10% of the molecular mass), and additionally by the polypeptide ubiquitin, and cytoskeletal proteins (medium and heavy chains of neurofilament polypeptides)^64^.

### Tau proteins

The tau protein was described almost half a century ago (1975) and identified as essential for “microtubules” (MTs) assembly^12^. This protein was initially observed in abundance in neurons, associated to MTs (microtubule-associated protein MAPT), which showed the ability to promote tubuline polymerization and MTs stability^12^. The tau protein was first identified as a component of the NFTs in AD brain using immunocytochemistry in 1986^65^.

**Figure 3:**
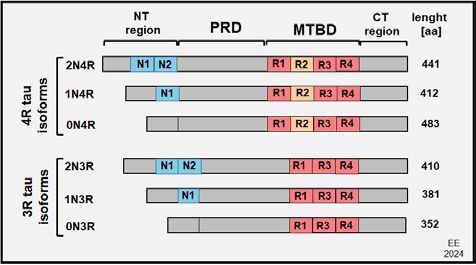
The six tau protein isoforms with four and three repeats.

Abbreviations: NT region, amino (NH2) terminus region; CT region, carboxy (COOH) terminus region; PRD, proline rich domain (functional structure with a role in the association of tau with actin); N1 and N2, N terminus inserts; MTBD, microtubule binding repeat domain; length [aa], amino acid length.

The human tau protein, encoded by the *MAPT* gene located on chromosome 17 (17q2), presents three different transcripts, one of them found mainly in the brain^66,67,68^. The results of processing the tau produce six isoforms: 0N/3R, 0N/4R, 1N/3R, 1N/4R, 2N/3R, and 2N/4R (where, R indicates the number of MT binding repeats, and N represents the number of N-terminal inserts). The mature human brain contains the 3R and 4R tau isoforms in approximately equal levels^66^, and the proportion of 1N (54%), 0N (37%), and 2N (9%) isoforms is expressed differentially within different neuronal types. The NFTs in AD contain both 3R and 4R isoforms, but different tau isoforms are overrepresented in pathological aggregates in other human tauopathies^45,67,69^ (Figure 3).

In normal conditions, tau proteins are mostly expressed in neurons and, to a lesser extent, in astrocytes and oligodendrocytes. In adult neurons, tau is predominant in axons, being present in the somatodendritic and also in the synaptic compartments of healthy neurons, contributing to synapse physiology^67,69^. In the axons, tau proteins interact, and upon binding, stabilizes the MTs directly or through acting as a cross bridge, which enables MTs to interconnect with other cytoskeletal components such as actin and neurofilaments^67,69^. This interaction regulates MTs assembly and stabilization, contributing to the structural elements of axons and facilitating the regulation of the transport of organelles and biomolecules to and from synapses^69^. The ability of tau to bind MTs is mediated by its MT-binding domains located in the C terminus. The N terminus extends from the MT region to facilitate its interaction with diverse components of the cytoskeleton or the plasma membrane^67,69^.

The tau proteins undergo many different post-translational modifications including phosphorylation, glycosylation, acetylation, nitration, methylation, prolyl isomerization, ubiquitylation, SUMOylation, glycation, and truncation. Such modifications can regulate tau binding to MTs, its metabolism, turnover, and aggregation. Phosphorylation is the most common post-translational modification of tau, and the first to be identified. It reduces tau binding to MTs and their stability. The detached tau monomers are highly soluble proteins which undergo self-assembly forming tau oligomers, can attach to other oligomeric structures, forming assembled β-sheet strings, and insoluble NTs and PHFs, which then turn into large NFTs^67,69,70,71^.

### Phosphorylated tau (phospho-tau)

The phosphorilation of tau can be performed through three classes of protein kinases: [1] proline-directed serine/threonine-protein kinases (e.g., GSK-3); [2] non-proline-directed serine/threonine-protein kinases, and [3] tyrosine kinases^71,72^. The phosphorylation state of tau is regulated by a balance between the activities of tau kinases and phosphatases, which thus regulate its function, with GSK-3β and PP2A playing prominent roles^67,69,71^.

Physiologically, tau is phosphorylated at several residues that regulate its association with MTs. However, in pathological states, specific sites on tau are aberrantly phosphorylated, which may decrease its association with MTs and increase its propensity to self-associate and form toxic oligomeric species^69^.

**Figure 4:**
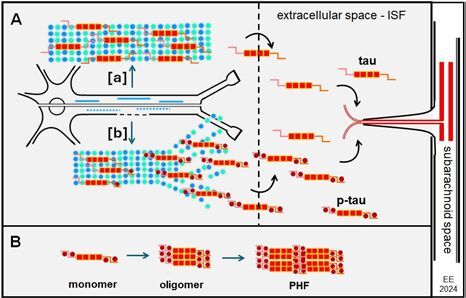
Tau pathways. **A.** Neuron with microtubules (blue). **[a]** Normal state – healthy neuron – arrow pointing to normal microtubule bounded by tau proteins. **[b]** Pathological state - impaired neuron – arrow pointing to disrupted microtubule due to presence of p-tau proteins. **B.** aggregation process of p-tau monomers and formation of paired helical filament (and next neurofibrillary tangles).

Abbreviations: ISF, interstitial fluid; PHF, paired helical filament.

Note: Extracellular space-ISF: locale where released tau and p-tau proteins reach the clearance pathways.

The tau protein contains 85 potential phosphorylation sites, and the main alterations of tau, under physiological conditions, are phosphorylation and dephosphorylation, which changes its association with MTs. Through regulation by phosphorylation, the major functions of tau proteins are to promote tubulin polymerization and to stabilize MTs. Once tau proteins are phosphorylated, forming p-tau, they are released from the MTs. The abnormally cytosolic p-tau proteins lose their ability to bind to normal MT-associated proteins, disrupting MTs assembly^67,73^. Additionally, tau controls the axonal transport of proteins and organelles by influencing the motor proteins dynein and kinesin. This process contributes to the degeneration of the affected neurons^69^ (Figure 4).

Structural biology studies have revealed that the dominant components of tangles in AD are PHFs and “straight filaments” (SFs), both types being composed predominantly of abnormally phosphorylated tau protein. The PHFs and SFs are also observed in NTs, which are neurites containing filamentous tau. The NTs apparently represent the major burden of tau in AD, and they are considered to originate from neurons containing NFTs^2,67^. Additionally, there are the NPs characterized by the presence of DNs processes filled with PHFs^2^.

### The tau release and clearance

Tau protein levels are high in the brain, plasma, and CSF of patients with AD, being measurable at these sites. It is also measurable in healthy individuals, which suggests that tau is released from neurons into the ISF independently of diseases. Thus, tau release may occur independent of neuronal death by an unconventional release pathway and is regulated by neuronal activation. Current information suggest that tau can be released through:

- Direct translocation across the plasma membrane;
- Membranous organelle-based unconventional secretion, and
- Shedding of plasma membrane-derived microvesicles^74^.

The cause of high tau levels in the brain and fluids in AD is not entirely clear. It could be triggered by several factors such as increased neuronal synthesis or release, decreased neuronal clearance, or impaired CSF clearance^75^. Most clearance of neuronal tau happens via catabolism rather than release into the CSF. Decreased clearance from the brain could also result from its inefficient degradation through the ubiquitin-proteasome system and/or autophagic-lysosomal degradation system^67,76^.

The abundance and distribution of NFTs can be characterized *in vivo* by tau-PET. Some fractions of p-tau in AD merge in the brain progressively and aggregate into insoluble filamentous deposits. On the other side, some soluble p-tau fractions are increasingly released into the CSF and blood (plasma), where they can be detected and quantified to provide indirect evidence of disease state^71,77^.

### Topography of cerebral tau

In AD, NFTs formed by PHFs of p-tau proteins are one of the components of the AT(N) classification, accepted for the description of multidomain biomarker findings for amyloid, tau, and neurodegeneration^55,56,57^.

The knowledge of the distribution patterns of the plaques is of great importance for a better understanding and interpretation of tau-PET images^14^.

The distribution pattern of the structures was obtained by Braak and Braak with silver staining techniques (Bodian, Bielschowsky, Campbell-Switzer, and Gallyas) or monoclonal antibody AT8^58,78^. Three kinds of lesion could be distinguished: NFTs, NTs, and NPs. The NFTs and NTs generally show a common and highly characteristic pattern of distribution, and because of its constancy, a distinction of stages is possible (Box 3).

**Box 3. Staging of neurofibrillary tangles/neuropil threads spreading according to Braak and Braak^58,78^.**

**TRANSENTORHINAL STAGES** [stages I and II ]

**Stage I.** Transentorhinal cortex: reduced number of NFTs and NTs; entorhinal cortex, and hippocampus (CA1): devoid or with isolated NFTs; BF and thalamus anterodorsal nucleus: absent or isolated NFTs.

**Stage II.** Transentorhinal cortex: numerous NFTs and NTs; entorhinal cortex and hippocampus (CA1): mild number of NFTs; magnocellular nuclei of BF and thalamus anterodorsal nucleus: mild changes.

**LIMBIC STAGES** [stages III and IV]

**Stage IlI.** Transentorhinal and entorhinal cortices: increased number of NFTs and NTs, and presence of “ghost tangles”; hippocampus (CA1): modest involvement; subiculum starting NFTs; BF: small number of NFTs; isocortex: incipient changes in basomedial areas.

**Stage IV.** Transentorhinal and entorhinal cortices: severely affected with NFTs and NTs, and large numbers of “ghost tangles”; hippocampus: CA1 - numerous NFTs, and CA4 - modest affection; subiculum: mild involvement; BF: moderate number of NFTs; isocortex: mildly affected; amygdala: many NFTs and NTs; claustrum: mildly affected; putamen and accumbens: may show NFTs; thalamus: reuniens nucleus moderately affected, and anterodorsal nucleus with dense NFTs and NTs.

**ISOCORTICAL STAGES** [stages V and VI]

**Stage V.** Transentorhinal and entorhinal cortices: severe load of NFTs and NTs, and numerous “ghost tangles”; hippocampus (CA1–CA4) and subiculum: numerous NFTs and NTs; fascia dentata: isolated NFTs; isocortex: severely affected; claustrum, and amygdala: affected; thalamus: anterodorsal nucleus with more pronounced alteration, and anteroventral nucleus with initial NFTs; hypothalamus (lateral tuberal nucleus): initial NFTs; SNpc: initial NFTs.

**Stage VI.** Transentorhinal and entorhinal cortices: changes more pronounced; hippocampus: numerous NFTs and NTs, and CA1 with severe loss of neurons, numerous NTs and “ghost tangles”; fascia dentata: small number of NFTs; subiculum:small number of NFTs, and numerous NTs; BF: large number of NFTs, and “ghost tangles”; isocortex association areas: severely affected; primary sensory areas: dense network of NTs, and small numbers of NFTs; primary motor field: reduced changes; amygdala: numerous NFTs and “ghost tangles”; thalamus (anteroventral nucleus and reticular nucleus): presenting changes; hypothalamus (lateral tuberal nucleus: with NFTs; striatum and SNpc moderately affected.

Abbreviations: NFTs, neurofibrillary tangles; NTs, neuropil threads; BF, basal forebrain; SNPc, substantia nigra pars compacta.

### Tau proteins as biological markers for Alzheimer’s Disease

The tau protein can be phosphorylated at different sites, as seen, comprising numerous amino acids (181, 199, 202, 205, 217, 231, 235, and 396). Several studies on CSF p-tau biomarkers detected p-tau217, p-tau231, and p-tau181 early in AD, and as the disease progressed, the other forms also became detectable. The findings also suggest that plasma p-tau231, p-tau217, and p-tau181 could be used interchangeably for clinical purposes^77^.

CSF p-tau181 and p-tau231 are well-established indicators of continuous tau pathology. Recent studies have also shown that p-tau217 may be more sensitive to AD diagnosis than p-tau181. Moreover, p-tau231 showed the strongest topographical associations with the earliest changes in Aβ-PET uptake compared to p-tau 217 and 181^79^.

Plasma levels of p-tau 181, 217, and 231 revealed age association, increased concentration with disease severity, and with intensity of Aβ and tau pathologies^79,80,81,82^.

## OTHER BIOLOGICAL MECHANISMS RELATED TO ALZHEIMER’S DISEASE

Inflammation is a key occurrence in AD pathogenesis, supported by several *in vivo* models and clinical studies. Patients with AD have high levels of inflammatory markers such as cytokines, chemokines, and acute-phase proteins in the brain and CSF, suggesting the presence of chronic low-grade inflammation^83,84,85,86^. Microglia and astrocytes are two types of glial cells related to AD pathogenesis. Aβ deposition can activate microglia, therefore culminating in an inflammatory response. The astrocytes have shown competence to clear Aβ peptides and their dysfunction has been linked to its accumulation in the brain^86^.

Oxidative stress occurs due to the imbalance between reactive species and antioxidant capability in cells. The structure, high lipid levels, and fast metabolism make the brain vulnerable to the damaging consequences of oxidative stress. When the antioxidant defense is unable to neutralize reactive species, mainly reactive oxygen species (ROS), this balance is interrupted, leading to oxidative stress, neuronal damage, and mitochondrial dysfunction, which are particularly vulnerable to oxidation due to their limited capacity to repair DNA. Mitochondrial dysfunction is also a common finding in AD and has been related to the accumulation of Aβ and NFTs. The Aβ peptide may lead to serious oxidation due to excessive ROS generation and decreased ATP production. Therefore, Aβ affects mitochondrial homeostasis, leading to impaired enzymatic activity and potential in the mitochondrial membrane^86,87,88,89^.

## THE INTERACTION BETWEEN THE DIFFERENT MECHANISMS

Interaction between Aβ and p-tau was found to be associated with a condition that could cause cognitive deficits in AD patients. Both hypotheses are interconnected and mutually reinforcing. Aggregates of Aβ peptides enhance the hyperphosphorylation of tau by mediating the activation of CDK-5. It was shown that natural Aβ oligomers, mainly dimers, are sufficient to accelerate hyperphosphorylation of tau at AD-relevant epitopes and cause instability in MTs cytoskeleton^77,90^. Another interaction is mediated by GSK-3 that has been proposed to function as a molecular link between Aβ and tau in AD pathogenesis — Aβ activates GSK-3 that in turn phosphorylates tau. On the other hand, GSK-3 regulates APP cleavage and Aβ production and promotes neuronal death induced by Aβ peptide^91,92^. Consequently, Aβ accumulation can induce tau hyperphosphorylation, thus leading to the formation of NFT. Furthermore, tau hyperphosphorylation can exacerbate Aβ deposition by impairing the clearance mechanisms of Aβ. This interplay between Aβ and NFTs promotes a synergistic effect, leading to neurodegeneration^93^.

Inflammation can also be triggered by Aβ plaques and, simultaneously, it contributes to Aβ accumulation and tau hyperphosphorylation. Pro-inflammatory mediators released by activated microglia can promote Aβ production, impair Aβ clearance, and induce tau pathology. The resulting neuroinflammation increases inflammatory responses, creating a cycle to exacerbate neuronal damage and disease progression^86,94^.

Oxidative stress can be induced by Aβ aggregation thus leading to ROS production and neuronal death. In turn, oxidative stress contributes to Aβ formation and tau hyperphosphorylation. Moreover, inflammation might exacerbate oxidative stress, further causing neuronal injury and dysfunction. Finally, dysfunctional mitochondria produce increased ROS and impair the proteolytic processing of APP, promoting increased Aβ production. Aβ can disrupt mitochondrial function by impairing mitochondrial dynamics through oxidative phosphorylation and membrane potential. Likewise, tau hyperphosphorylation may affect mitochondrial dynamics, bioenergetics, and transport, contributing to mitochondrial dysfunction^86,95^.

In conclusion, this paper provides a review about the major hypotheses of AD pathogenesis, with highlights of biological mechanisms and their potential interplay. There is not only one mechanism related to AD. This is confirmed by recent studies that have made significant advances in elucidating the underlying molecular mechanisms of this disease and their interconnection.

The basis for understanding the source of biological markers was also clarified. Such markers can be detected through fluid dosages (blood plasma and CSF), and PET images labeled for Aβ and tau. Such resources allow for early diagnosis of AD, as well as the correct selection of patients to receive the new treatment strategies that are in advanced stages of development.
